# Comparison of burden among family members of patients diagnosed with schizophrenia and bipolar disorder in a large acute psychiatric hospital in China

**DOI:** 10.1186/s12888-016-0962-y

**Published:** 2016-08-11

**Authors:** Yanling Zhou, Robert Rosenheck, Somaia Mohamed, Yufen Ou, Yuping Ning, Hongbo He

**Affiliations:** 1 Department of Psychiatry, Guangzhou Brain Hospital, (Guangzhou Huiai Hospital, The Affiliated Brain Hospital of Guangzhou Medical University), #36, Mingxin Road, Liwan District, Guangzhou, Guangdong Province 510370 China; 2 Department of Psychiatry, Yale university school of medicine, New Haven, CT USA

**Keywords:** Family burden, Caregiver, Schizophrenia, Bipolar disorder, Predictor

## Abstract

**Background:**

The difference of burden between caregivers of acute patients with schizophrenia and bipolar disorder has not been well studied in China, a culture where family responsibility has a very high value. Our aim is to compare family burden in these two categories diagnosis and to identify predictors of family burden in a large psychiatric hospital in China.

**Methods:**

Two hundred forty-three schizophrenic patients and 200 bipolar patients were enrolled in a cross-sectional study. Patients were independently evaluated on symptoms, insight, attitudes toward medication, quality of life during the first week of their admissions. The prime caregiver for each patient was also evaluated with a standard measure of family burden within 1 week of patients’ admission.

**Results:**

Caregiver perceptions of violent behavior and suicidal risk among patients with bipolar disorder were significantly greater than among families of those with schizophrenia. Hierarchical regression analyses demonstrated differential correlates of burden for all predictive factors with R^2^ values ranging from 0.14 to 0.27 in the five burden factors in schizophrenia families; and from 0.12 to 0.24 in bipolar disorder families. Symptoms severity explained the greatest proportion of variance, whereas patient and caregiver demographic variables explained much less variance.

**Conclusion:**

Family burden, especially the caregiver perceptions of violent and suicidal behaviors were greater in care givers of acute bipolar disorder patients than among caregivers of schizophrenia patients in the present sample. However, in families of patients with both disorders clinical features were the strongest predictor of caregiver burden.

## Background

Schizophrenia and bipolar disorder are two of the most serious and debilitating psychiatric disorders, each affecting approximately 1 % of the population worldwide [1]. For those living with a mental illness, especially in the developing world in which the availability of community support service is limited, family members are the major, if not the exclusive, sources of support and care.

Family caregivers shoulder the vast majority of long-term care responsibilities worldwide without pay or compensation [2]. Caregiver studies have documented numerous adverse effects of caregiving for a mentally ill family member, impairing quality of life, causing time lost from work, financial stresses, limiting time for leisure and socializing, as well as causing adverse health effects such as elevated stress and depression, feelings of stigmatization, poorer self-rated health, chronic medical conditions, greater use of tranquilizers and antidepressants and increased risk of medical hospitalization [3–5].

Severe mental illness has been found to affect caregivers in complex ways. Disability, impaired functioning and symptom severity of the ill family member have all been identified as predictors of consequences for caregivers [6, 7]. Moreover, the characteristics of caregivers themselves, and of their relationship to patients, may also be important determinants. For instance, being older, being a parent, or spending increasing numbers of hours caring for the patient may increase burden [8]. Ample social support and adaptive coping, in contrast, may be protective (e.g., problem-solving seems more effective than avoidance or other emotional coping strategies) [9]. Finally, regarding the well-known association between caregiver burden and patient distress, the direction of causality remains a matter of debate [8, 9].

China is a middle income country and the special social, cultural and economic factors lead family members have to take primary responsibility for the provision of care for other family member with a mental illness. Firstly, Chinese health beliefs derives its origin from the three pillars of Chinese philosophy, including Confucianism, Buddhism and Taoism, which influence most of caregiver, particularly parents, with a belief that they have to take care of their sick family member with as much time and effort as possible [10, 11]. Secondly, community mental health services in China remain limited and majority of caregivers cannot receive any support from public social services [11, 12], which might enhance caregiver burden. Thirdly, the economic burden of mental illness was serious in China, especially the cost of in-patients is higher than the cost of out-patients, because health insurance coverage remains inadequate for many people and out-of-pocket expenditures remain high [13].

Research methods have been developed in the last two decades to assess family stress and burden among caregivers of patients diagnosed with schizophrenia and bipolar disorder. However, comparison of the type and relative severity of family burden for hospitalized patients with schizophrenia and bipolar has not been studied, especially in China. In this study we compared family burden among the primary caregiver of patients in a large psychiatric hospital in Guangzhou China for either acute schizophrenia or acute bipolar disorder.

The principal hypotheses tested here was that families will experience different kinds of burden according the diagnosis of their family member. A secondary goal was to examine the impact of various aspects of schizophrenia and bipolar disorders through cross-sectional analyses of the associations of patient and caregiver characteristic with the type and severity of experienced burden.

## Methods

### Subjects

The Guangzhou Brain Hospital (GBH) is a 1900-bed acute psychiatric hospital in Guangzhou, China, China’s third largest city. Recruitment took place from June 2012 to December 2013. Five hundred eighty consecutive admissions to nine psychiatric units were recruited for the study according to the following criteria: age (16–60 years), having diagnosis of schizophrenia (*n* = 328) or bipolar disorder (*n* = 252) as determined by the Diagnostic and Statistical Manual of Mental Disorders, 4th edition (DSM-IV). Exclusion criteria included dementia and debilitating medical illness. Subjects were excluded if they had confusional state, psychosis of organic origin, or did not speak Chinese. Patients who admitted to the hospital were invited to be evaluated within seven days of admission and their primary caretakers (the family member who spent the most time with the patient) were also invited to participate in a survey of family burden. The interview covered the month preceding admission to the hospital. Eligible subjects were judged by the investigators and all subjects including patients and caregivers gave written informed consent. The authorization for the study was obtained from the Guangzhou Brain Hospital Ethics Committee (equivalent to an Internal Review Board, elsewhere).

A total of 328 schizophrenic patients and 252 bipolar subjects were assessed during the recruitment phase. 243 subjects with schizophrenia and 200 with bipolar disorder were examined together with their caregivers. In 137 cases, the caregivers did not participate for the following reasons: either the patients themselves or their families refused, the patient could not name any caregivers, or it was not possible to contact them. We compared demographics data including age, gender, education, employment status, marital status, insurance coverage, duration of illness and number of previous admission from those 443 patients and those whose caregiver did not participate in burden interview and found that there was no significant statistical difference (*P* > 0.05).

### Measures

#### Patient measures

Symptoms of schizophrenia were assessed using the Positive and Negative Syndrome Scale (PANSS) with scores ranging from 30 to 210, with higher scores indicating more severe symptoms [14].

Severity of mania and depression symptoms of bipolar disorder was assessed using the Young Mania Rating Scale (YMRS) and the 17-item version of the Hamilton Depression Rating Scale (HAMD-17), respectively.

Insight was assessed using the Insight and Treatment Attitudes Questionnaire (ITAQ). This well-validated, structured interview [15], includes 11 questions assessing recognition of mental disorder and attitudes towards the need for medication, hospitalization, and follow-up care.

Before the study, an inter-rater reliability exercise of PANSS, HAMD, YMRS and ITAQ was conducted on 10 patients with symptomatic schizophrenia and 10 patients with affective disorder. Assessment of inter-rater reliability for nine raters in this study was in the excellent to good range for all the scales used, with intra-class correlation for the PANSS, HAMD, YMRS and ITAQ total score was 0.92, 0.88, 0.92, and 0.88 respectively.

Attitudes toward medication were assessed by the Drug Attitude Inventory (DAI) [16] a ten-item true-false scale, on which higher numbers indicate more positive views toward medication. The instrument focuses on unpleasant and negative subjective responses that are common adverse effects of antipsychotic medications.

Quality of life was evaluated by the Short-Form Health Survey (SF-36) Chinese version [17]. SF-36 scales measure perceived health in the areas of physical functioning (PF), role physical (RP), bodily pain (BP), general health (GH), vitality (VT), social functioning (SF), role emotional (RE), and mental health (MH), with higher scores reflecting better perceived health. The eight multi-item scales are aggregated into the physical component summary (PCS) and mental component summary (MCS), but with higher weights for the first four scales in the PCS and for the last four scales in the MCS.

### Caregiver measures

Family burden was assessed by the Family Experience Interview Schedule (FEIS) [18, 19], which was used in the Clinical Antipsychotic Trials of Intervention Effectiveness (CATIE) study similarly. We had assessed the validity and reliability of a 28-item brief adapted Chinese version of the FEIS among caregivers of patients with mental disorders. We identified the five dimensions using exploratory and confirmatory analyses: Factor 1 indicates violent behavior of the patient as perceived by the caregiver (almost always the family); Factor 2 reflects depression and anxiety symptoms and social isolation of the caregiver; Factor 3, reflects disruption of caregiver routines because of a need to take care of the patient’s daily life needs and problematic behaviors; Factor 4, reflects caregiver apprehensions about patient suicidality; Factor 5 reflects satisfaction with the quality of service provision as perceived by the caregiver. These five diemnsions had good internal consistency and, thus, appeared to assess valid dimensions of family burden in Chinese care-givers of persons with serious mental illnesses [20].

Within each burden factor, because the units used to measure the items are different, the factor score have to undergo normalizing transformation before further analysis.

### Data analysis

First, to examine differences in the sociodemographic and clinical characteristics of patients, and the sociodemographic characteristics of caregivers between the two diagnostic groups using t-tests for continuous variables and Chi-square tests for categorical variables were applied.

Next, we compared family burden data from schizophrenia and bipolar disorder on burden factors after the normalization. Stepwise linear regression analysis was used to investigate the independent relationship between the five family burden factors and diagnosis net of other patient and caregiver demographic variables, insight, attitudes toward medication, quality of life.

Finally, the proportion of explained variance of the family burden was investigated using multiple regression analyses.

Patient and caregiver demographic variables and clinical features were entered into five hierarchical regression analyses to explore associations of each burden factor. Similar conceptual variables were given in a same group and then were entered into the regression model in a single step. Patient demographic variables were entered in step 1, and then caregiver demographic variables and clinical features were entered in step 2 and 3.

Statistical analyses were performed using the Statistical Package for the Social Sciences (SPSS) version 17.0. The level of significance was set at a = 0.05.

## Results

### Sample characteristics

There was significant difference in age of patient (mean = 35.5 years, SD = 12.2 for schizophrenia and 31.3 years, SD = 12.3 for bipolar disorder; t = 3.575, *p* < 0.001). Significant difference in insurance was also noted between the schizophrenia group and bipolar disorder samples, with the schizophrenia sample including more patients covered by insurance (43.6 %, *N* = 106 vs. 34.0 %, *N* = 68, *x*^*2*^ = 4.258, *p* = 0.039). In subjects with schizophrenia, the average total PANSS score was 93.5 (SD = 15.1); the average total HAMD score was 21.6 (SD = 11.2) and the average total YMRS score was 31.6 (SD = 11.7) in subjects with bipolar disorder. There was no significant difference in ITAQ, DAI, SF-36, gender, duration of illness, marital status, number of previous admission as well as other sociodemographic and clinical patient characteristics (Table 1).

**Table 1:** Comparison of patients diagnosed with schizophrenia and bipolar disorder on social-demographic characteristics, ITAQ, DAI, SF-36 scores with t-tests or chi-square test

Variables	SP (N=243)		BD (N=200)		Statistics	*p*
	Mean/N	S.D./%	Mean/N	S.D./%		
Patient’s characteristics						
Age(years)	35.5	12.2	31.3	12.3	t=3.575	<0.001^***^
Onset age	26.5	9.8	25.9	9.7	t=0.661	0.509
Duration of illness(year)	8.5	8.9	8.1	8.5	t=0.522	0.602
Education (years)	11.3	3.0	11.4	3.5	t=−0.601	0.548
Gender (male)	136	56.0%	98	49.0%	*x* ^*2*^=2.137	0.086
Employed	59	24.3%	55	27.5%	*x* ^*2*^=0.595	0.440
Positive family history	67	27.6%	60	30.0%	*x* ^*2*^=0.316	0.574
Number of previous admissions		*x* ^*2*^=2.025	0.567			
0	77	31.7%	62	30.1%		
1	57	23.6%	49	24.5%		
2	36	14.8%	38	19.0%		
≥3	73	30.0%	51	25.5%		
Marital status		*x* ^*2*^=1.855	0.396			
Unmarried	131	53.9%	116	58.0%		
Married	91	37.4%	73	36.5%		
Divorce/widow	21	8.6%	11	5.5%		
With insurance	106	43.6%	68	34.0%	*x* ^*2*^=4.258	0.039^*^
Patient’s clinical features						
PANSS positive	24.1	6.4	--	--		
PANSS negative	24.0	7.7	--	--		
PANSS general	45.5	8.2	--	--		
PANSS total	93.5	15.1	--	--		
HAMD total	--	--	21.6	11.2		
YMRS total	--	--	31.6	11.7		
ITAQ total	5.6	5.2	6.5	5.9	t=−1.813	0.070
DAI total	1.2	5.3	1.3	5.2	t=−0.160	0.873
SF-36 PCS	47.8	31.8	53.7	33.3	t=−1.707	0.089
SF-36 MCS	48.3	30.5	48.8	30.3	t=−0.135	0.892

Abbreviations: *SP* Schizophrenia, *BD* Bipolar Disorder, *PANSS* Positive and Negative Syndrome Scale, *HAMD* Hamilton Depression Rating Scale, *YMRS* Young Mania Rating Scale, *SF-36 PCS* Short Form 36 Health Questionnaire Physical Component Summary, *SF-36 MCS* Short Form 36 Health Questionnaire Mental Component Summary, *ITAQ* Insight and Treatment Attitudes Questionnaire, *DAI* Drug Attitude Inventory, *S.D.* Standard Deviation, *N* Number

Reported correlations are statistically significant at the *p < 0.05 level; or ***p < 0.001

There was significant difference in age of caregiver (mean = 47.7 years, SD = 13.7 for schizophrenia and 44.4 years, SD = 13.6 for bipolar disorder; t = 2.531, *p* = 0.012) as well as in frequency of contact in the past month: contact frequency with caregivers of patients with schizophrenia was less than that in caregivers of bipolar disorder (*x*^*2*^ = 13.638, *p* = 0.003). There was no significant difference in marital or employment status, or relationship to patient of caregiver between two groups (Table 2).

**Table 2:** Comparison of patients diagnosed with schizophrenia and bipolar disorder on caregiver’s characteristics with t-tests or chi-square tests

Variables	SP (*N*=243)		BD (*N*=200)		Statistics	*p*
	Mean/N	S.D./%	Mean/N	S.D./%		
Age (years)	47.7	13.7	44.4	13.6	t=2.531	0.012*
Relationship to patient					*x* ^*2*^=1.716	0.633
Parent	115	47.3%	104	52.0%		
Child	27	11.1%	16	8.0%		
Spouse	50	20.6%	38	19.0%		
Other	51	21.0%	42	21.0%		
Live with patient in the past month	182	74.9%	149	74.5%	*x* ^*2*^=0.009	0.999
Live with patient at any time during the past month	154	63.4%	121	60.5%	*x* ^*2*^=0.385	0.535
Employed	131	53.9%	96	48.0%	*x* ^*2*^=1.533	0.216
Marital status					*x* ^*2*^=4.518	0.104
Unmarried	43	17.7%	22	11.0%		
Married	181	74.6%	165	82.5%		
Divorce/widow	19	7.8%	13	6.5%		
Contact with patient in the past month					*x* ^*2*^=13.638	0.003**
Not at all	8	3.3%	14	7.0%		
Once	11	4.5%	15	7.5%		
Once (or more) per week	49	20.2%	60	30.0%		
Once (or more) per day	175	72.0%	111	55.5%		

See Table 1 for abbreviations

Reported correlations are statistically significant at the *p < 0.05 level; or **p <0.01

### Comparison of burden factor

Stepwise linear regression analysis showed that caregiver perceptions of violent behavior and suicidal risk among caregivers of patients with bipolar disorder group were greater than for caregivers of patients with schizophrenia (violent behavior: B = 2.013, *p* < 0.001; suicidal behavior: B = 0.506, *p* = 0.030) (Table 3). There were no significant differences on factors representing caregiver distress, disrupted routines or satisfaction with services.

**Table 3:** Results of the stepwise regression analysis of the five family burden factors and characteristics and clinical features (*N*=443)

Dependent variables	Independent variables	B	S.E.	Beta	t	*P*	95% C.I. for B	
							Lower	Upper
Factor 1								
	ITAQ	−0.204	0.039	−0.234	−5.195	<0.001	−0.281	−0.127
	Diagnosis (BD vs SP)	2.013	0.434	0.209	4.638	<0.001	1.160	2.866
	Female (patient)	−1.353	0.435	−0.141	−3.113	0.002	−2.207	−0.499
	Employed (patient)	−1.319	0.494	−0.120	−2.668	0.008	−2.290	−0.347
	Employed (caregiver)	−0.965	0.432	−0.101	−2.233	0.026	−1.815	−0.116
Factor 2								
	ITAQ	−0.014	0.007	−0.096	−2.029	0.043	−0.028	0.000
Factor 3								
	Employed (caregiver)	1.370	0.456	0.140	3.006	0.003	0.474	2.266
	Duration of illness	0.079	0.026	0.140	3.030	0.003	0.028	0.129
	Female (patient)	−1.309	0.456	−0.133	−2.873	0.004	−2.204	−0.414
	SF-36 MCS	−0.016	0.008	−0.091	−1.970	0.049	−0.032	0.000
Factor 4								
	Age	−0.038	0.009	−0.189	−4.000	<0.001	−0.056	−0.019
	Female (patient)	0.539	0.233	0.109	2.317	0.021	0.082	0.996
	Diagnosis (BD vs SP)	0.506	0.233	0.102	2.176	0.030	0.049	0.963
	Divorce/widow (caregiver)	1.008	0.443	0.105	2.277	0.023	0.138	1.878
	Employed (caregiver)	−0.504	0.231	−0.102	−2.187	0.029	−0.958	−0.051
Factor 5								
	ITAQ	0.051	0.020	0.120	2.557	0.011	0.012	0.090
	Age	0.019	0.009	0.100	2.124	0.034	0.001	0.036

See Table 1 for abbreviations

*Factor 1* violent behavior, *Factor 2* caregiver distress, *Factor 3* disrupted routines, *Factor 4* suicidal behavior, *Factor 5* satisfaction with services

### Predictors of burden factor

Because diagnosis proved to be associated with some measures of caregiver burden an attempt was made to find factors that better predicted the burden in schizophrenia and bipolar groups respectively. For this purpose, multiple regression analyses for each of the five burden factors on the FEIS were performed with the characteristics of the patients, caregivers and clinical features entered sequentially into the model. For simplicity of presentation, only the third step, which controlled for the effects of all other variables, is presented.

Table 4 presents the results of the multiple regression analyses for the five burden factors in the schizophrenia sample. The model of burden in the primary caregivers of patients with schizophrenia revealed R^2^ values ranging from 0.147 for satisfaction with services to 0.268 for violent behavior of the patient as perceived by the caregiver. The set of patient’s clinical features explained the largest proportion of the observed family burden variance in all its dimensions i.e. 13.6 for violent behavior, 9.0 for caregiver distress, 8.9 for disrupted routines, 5.8 for suicidal behavior, and 8.4 % for satisfaction with services. Clinical patient characteristics had lesser influence on the variance of the family burden factors ranging from only 3.3 % to 8.5 % across factors. Characteristics of the caregiver had even lower association with burden scales with the explained variance of less than 5 %.

**Table 4:** Results of the stepwise regression analysis of the family burden factors in the group of patients diagnosed with schizophrenia and the three sets of patient and caregiver characteristics and patient clinical features (*N*=243)

Independent variables	Violent behavior	Caregiver distress	Disrupted routines	Suicidal behavior	Satisfaction with services
Patient’s characteristics					
Age			−0.105*	−0.045*	
Gender	−2.102**			0.822*	
Education		0.206*			
Duration of illness			0.008*		
Carer’s characteristics					
Age		−0.042*			
Child			−2.979*		
Patient’s clinical features					
PANSS positive	0.102**		0.299*		
PANSS negative			0.185*		
PANSS general		0.221**		0.003*	
ITAQ total	−0.228***				−0.082*
DAI total		−0.159**	−0.414*	−0.120*	
SF-36 MCS					−0.016*
Change in R^2^ by step					
Patient characteristics	0.085	0.033	0.046	0.048	0.048
Care’s characteristics	0.047	0.018	0.026	0.044	0.015
Patient’s clinical status	0.136	0.090	0.089	0.058	0.084
Total R^2^	0.268***	0.141*	0.161*	0.150*	0.147*

See Table 1 for abbreviations

Reported correlations are statistically significant at the *p < 0.05 level; or **p <0.01; or ***p < 0.001

Similarly, in bipolar disorder group, the model for each of the five burden factors had R^2^ values ranging from 0.177 for caregiver distress to 0.236 for violent behavior of the patient as perceived by the caregiver. The set of patient clinical features explained the largest proportion of the variance in observed family burden accounting for 10.6 of violent behavior, 5.2 of caregiver distress, 10.3 of disrupted routines, 8.9 of suicidal behavior, and 5.2 % of satisfaction with services. Characteristics of patient and caregiver explained had even smaller proportion of variance ranging from 3.2 % to 11.7 % (Table 5).

**Table 5:** Results of the stepwise regression analysis of the family burden factors in patients diagnosed with bipolar disorder and the three sets of patient and caregiver characteristics and patient clinical features (*N*=200)

Independent variables	Violent behavior	Caregiver distress	Disrupted routines	Suicidal behavior	Satisfaction with services
Patient’s characteristics					
Age				−0.077*	
Gender			−1.850*		
Carer’s characteristics					
Age		−0.069*			
Spouse	2.156*			1.252*	1.752*
Divorce /widow					−1.992*
Patient’s clinical features					
HAMD total		0.304*		0.417**	
YMRS total	0.110**		0.395*		
ITAQ total	−0.174**				
DAI total					−0.074*
SF-36 MCS					−0.015*
Change in R^2^ by step					
Patient’s characteristics	0.080	0.033	0.037	0.060	0.061
Carer’s characteristics	0.050	0.032	0.068	0.051	0.117
Patient’s clinical features	0.106	0.052	0.103	0.089	0.052
Total R^2^	0.236**	0.117*	0.208*	0.200*	0.230*

See Table 1 for abbreviations

Reported correlations are statistically significant at the *p < 0.05 level; or **p <0.01

## Discussion

This study showed that burden was greater in caregivers of acute bipolar disorder patients than among caregivers of schizophrenia patients. Through use of multivariate modeling, we were able to demonstrate that different aspects of family members’ experiences supporting the acute phase of a relative with schizophrenia and bipolar disorder were associated with different dimensions of the patient’s clinical presentation, patient’s and caregiver’s characteristics. Each of the five burden factors was associated with a different pattern of relationships to the domains examined. However, patient’s clinical presentation was better predictor for caregiver burden in the acute phase in both diseases.

### Comparison of family burden

Despite an encouraging number of studies investigating family burden in caregivers of persons with severe mental illness, few studies had compared differences between caregivers of patients with schizophrenia and bipolar disorder. Some reported that caregivers of bipolar disorder patients suffered the similar degree of burden and cost of care as caregivers of schizophrenia [21, 22], whereas others suggested that the burden was heavier on the caregivers of schizophrenia [23, 24]. However, these studies mainly focused the caregiver burden in clinically stable outpatients, and the results might not be generalized to patients with acute exacerbation. Our study was based on personal interviews with a large sample of caregivers of schizophrenia and bipolar disorder. The present results were inconsistent with the observation of some others in that we found caregivers of patients with schizophrenia and bipolar disorder differed significantly with respect to several dimensions of perceived family burden with greater burden in bipolar disorder. Furthermore, we found that family burden, caregiver perceptions of violent and suicidal behavior were especially greater in caregivers of acute bipolar disorder patients than among caregivers of schizophrenia patients.

There was no significant difference between the two groups on socio-demographic variables like gender, relationship between patient and caregiver, duration of illness and number of previous admissions, indicating that the difference in the caregiving experience between the two groups could not be accounted for by these variables. The schizophrenia group was older, more likely to be covered by insurance and had closer contact with their caregivers than the bipolar caregiver group, but analysis of covariance analysis was used to adjust for the effect of these variables.

In terms of clinical severity, although schizophrenic patients and bipolar patients were assessed by different symptom scale, with an average total PANSS score of 93.5, subjects with schizophrenia showed a severe of symptoms according to a recently published set of severity standards [25]; and in subjects with bipolar disorder, the average total HAMD score was 21.6 and the average total YMRS score was 31.6 indicated that they presented with moderate severity of depressive symptoms and severe manic symptoms. There was no significant difference between the patient groups on ITAQ, DAI and SF-36, indicating that schizophrenic patients had insight, attitudes towards medication and quality of life levels not dissimilar from those with bipolar disorder in the acute phase, a finding that was consistent with previous studies [26–28].

Caregivers of patients with schizophrenia and bipolar disorder perceived similar distress, disrupted routines and satisfaction with services, a finding that was similar to earlier research. This suggested that, overall, both disorders had a similar impact on the family and there was similar need for caregiver support. But in the aspects of violent behavior and suicidal behavior, caregivers of patients with bipolar disorder perceived the burden to be heavier compared to caregivers of patients with schizophrenia. These results were different from those of most of the previous research which found that no differences in caregiver burden were found between the diagnostic groups [8, 21], or caregivers of schizophrenia patients experienced more burden than those of bipolar disorder patients [23, 24]. Although we had no conclusive explanation for the difference in results between these studies and the present one, there were several possibilities. The most likely cause of these inconsistencies was that the subjects in our study were acutely hospitalized, while those in other studies appear to have been clinically stable outpatients living in the community. From the regression analyses presented in Table 4 and 5, we saw that psychotic symptoms were associated with violent and suicidal behaviors as perceived by caregivers in the schizophrenia group, and similarly, clinical symptoms were associated with burden factors in the bipolar group, suggesting that diagnosis was a predictor of caregiver burden because of diverse acute symptoms. However since different symptom measures were used in the two groups we could not compare them on overall symptom severity. Secondly, it was also possible that methodological differences of family burden measurement between our study and others might lead to different results. The FEIS instrument included items which evaluated patient problem behavior, activities of daily living, role functioning and many sources of disruption for the family from the patient’s illness. The measure itself might be more sensitive to certain problem behaviors (e.g. violent and suicidal behaviors) characteristic of bipolar patients than evaluation instruments used in previous studies.

#### Predictors of family burden

Since diagnostic groups featured a different level of the burden, we explored correlates of burden in the two diagnostic groups. The clinical correlates can be viewed as conditions that were conducive to stress: including the functional status of the ill relative, the course and prognosis of the illness, various symptoms and behavioural problems [29]. The view that the clinical characteristics, functional status, and current symptomatology would predict caregiver stress was partially supported. Regression analyses showed that the patient’s clinical feature indeed best explained the observed variance in the FEIS factors as contrasted to the patient’s characteristics which explained no more that 8.5 % of the variance although caregiver characteristics explained 11.7 % variance for satisfaction with services in bipolar disorder group.

Large literatures have reported the association between family burden and symptom in serious mental illness, but the results were inconsistent. In subjects with schizophrenia in our study, although caregivers’ experiences of violent behavior were associated with positive symptoms, their experience of distress and suicidal behavior was associated with general psychopathology symptoms and their experience of disrupted routines was associated with positive and negative symptoms. In Perlick’s report, the disruption of routine was positively related to both positive and negative symptoms, whereas the problem behavior was positively associated with positive symptoms alone, the impairment in activities of daily living was positively associated with negative symptoms [19]. Mui found that increased burden was associated with higher levels of negative symptoms alone in older patients [30] and Magliano and Wolthaus found that only with positive symptoms [31, 32]. The most likely cause of such inconsistencies may be due to methodological differences of burden measurement which might focus on different dimensions of burden. Secondly, different homogeneous sample in different studied might cause this discrepancies, such as in- patients in present study, out-patients in Perlick’s [19].

In subjects with bipolar disorder, although relatives’ experiences of violent behavior and disrupted routines were associated with mania symptoms; and their experience of distress and suicidal behavior was associated with depressive symptoms. These showed that different aspects of burden caregiver perceived might be affected by different symptoms of patients with bipolar disorder, just like those with schizophrenia. Mueser et al. found that bipolar patient with more serious manic symptoms predicted more family burdensome [33]. Perlick et al. indicated that level of burden increased in relation to the presence of depressive symptoms on the subjective problem behaviour scale, but was not significantly related to the presence of psychoticism on any burden index [34]. Hooley et al. found negative or depressive symptoms to be more strongly associated with relative distress than positive symptoms [35].

Our findings indicated that the patient’s insight and attitude toward medication also contributed to the caregiver’s perceived burden, similarly in the two different regression models. Perlick et al. also pointed out that lower insight and more negative attitudes toward medication among schizophrenia patients predicted higher family burden, using the same assessment instruments as in our study [19].

Quality of life was positively associated with satisfaction with mental health services not only in schizophrenia but also in bipolar disorder. It was unexpected that patient clinical symptom severity assessed by PANSS, HAMD or YMRS showed no significant correlation with caregiver satisfaction with services, suggesting that even when the more florid symptoms of illness had been controlled, caregivers continued to be concerned about the patient’s ability to achieve the normal satisfaction with social life, work life, and leisure activities.

Some interesting findings emerged out of this study. It was clearly seen that the extent and pattern of family care burden among families of bipolar disorder was more severe than that among primary caregivers of schizophrenia. Furthermore, this study could provide a few clues that family burden might be predicted by patient’s clinical feature more than patient and caregiver demographic variables in acute phase. However, the variances of clinical feature explained caregiver’s burden were relatively small. Thus, we considered that other important factors might predict family burden such as drug side effect, cognitive function and social ability of patient [19], economic status [36], psychology of caregiver, which had so far failed to properly assess in this study. Clinical interventions for families were most often delivered when the patient was acutely ill, often as part of hospital stay. Our results underscored the equal or even greater need for intervention with family members of bipolar disorder as those of schizophrenia. Adequacy of family supports and educational interventions are needed to reduce caregiver burden in such serious mental illness.

### Limitations

The results were limited by the cross-sectional analyses employed, which precluded us from making inferences about causality. In addition, because only the primary caregiver was studied, we cannot draw inferences about the nature or level of burden experienced by other family members. Thirdly, the factors that produced burden were more present in this sample in acute bipolar than in acute schizophrenia, and the results could not represent stable patients in community. Finally, the two groups of patient could not be compared on overall symptom severity in Table 1, because symptoms were measured by using different disease-specific measures: the PANSS in the schizophrenia and the HAMD and YMRS in the bipolar.

## Conclusions

Family burden, especially the caregiver perceptions of violent and suicidal behavior were greater in care givers of acute bipolar disorder than among caregivers of schizophrenia. However, patient symptom severity was a better predictor of caregiver burden in the acute phase in both diseases. Further study is needed of factors that can minimize family burden.

## Abbreviations

DAI, Drug Attitude Inventory; DSM-IV, Diagnostic and Statistical Manual of Mental Disorders, 4th edition; FEIS, Family Experience Interview Schedule; GBH, Guangzhou Brain Hospital; HAMD, Hamilton Depression Rating Scale; ITAQ, Insight and Treatment Attitudes Questionnaire; MCS, Mental Component Summary; PANSS, Positive and Negative Syndrome Scale; PCS, Physical Component Summary; S.D., Standard Deviation.; SF-36, Short Form 36 Health Questionnaire; YMRS, Young Mania Rating Scale
